# Combined effects of pioglitazone and doxorubicin on migration and invasion of MDA-MB-231 breast cancer cells

**DOI:** 10.1186/s43046-022-00110-x

**Published:** 2022-03-28

**Authors:** Parisa Malakouti, Mobin Mohammadi, Mohammad Amin Boshagh, Abbasali Amini, Mohammad Ali Rezaee, Mohammad Reza Rahmani

**Affiliations:** 1 Student Research Committee, Kurdistan University of Medical Sciences, Sanandaj, Iran; 2 Department of Immunology and Hematology, Faculty of Medicine, Kurdistan University of Medical Sciences, Sanandaj, Iran; 3 Breast Cancer Research Center, Motamed Cancer Institute, ACECR, Tehran, Iran; 4 Department of Medical Laboratory Sciences, Faculty of Paramedical, Kurdistan University of Medical Sciences, Sanandaj, Iran

**Keywords:** Breast cancer, Doxorubicin, Pioglitazone, CXCR4, CXCR7

## Abstract

**Background:**

Despite antitumor properties, chemotherapy medication can create conditions in tumor cells that work in favor of the tumor. Doxorubicin, commonly prescribed chemotherapy agents, can increase the risk of migration and invasion of tumor cells through overexpression of the CXCR4 gene by affecting downstream signaling pathways. The regulatory role of CXCR7 on CXCR4 function has been demonstrated. Therefore, it is hypothesized that combining doxorubicin with another anticancer drug could be a promising approach.

**Methods:**

In this research, we evaluated the anti-invasive property of pioglitazone along with antitumor effects of doxorubicin on MDA-MB-231 breast cancer cell lines.

**Results:**

There was no significant difference between two treatment groups in neither the expression nor changes in the expression of CXCR7 and CXCR4 genes (*P* < 0.05). Pioglitazone-doxorubicin combination reduced cell migration in tumor cells to a significantly higher extent compared to doxorubicin alone (*P* < 0.05).

**Conclusions:**

Co-administration of pioglitazone and doxorubicin might reduce cell migration in breast cancer tumor cells, and that cell migration function is independent of some specific proteins.

**Supplementary Information:**

The online version contains supplementary material available at 10.1186/s43046-022-00110-x.

## Background

Breast cancer accounts for 25% of all cancers and is the most common malignancy among women globally [1]. It was estimated that 280,000 new cases of invasive breast cancer diagnosis are made among American women by the end of 2021 [2]. Occurrence of metastatic breast cancers is considered as a reason for treatment failure and death despite the factors attributed to the prognosis of primary breast cancers. Triple-negative breast cancer (TNBC) is an advanced breast cancer that is associated with high metastatic potential and mortality rate and poor prognosis compared to other types of breast cancer [3, 4]. Metastasis is a function of several successive stages of highly scheduled and non-random processes. Chemokines and their receptors play an essential role in all steps of tumor progression [5]. CXCR4 is one of the most frequently expressed chemokine receptors on the surface of tumor cells [6]. CXCR4-CXCL12 pathway facilitates the implantation and metastasis in CXCL12-producing organs including the liver, bone marrow, brain, heart, and lungs [7, 8]. However, after binding to CXCL12, CXCR4 is rapidly phosphorylated and is destroyed in the lysosome after entering the cell [9, 10]. It is believed that chemotaxis in breast cancer cell does not depend on a fixed level of CXCL12 but is stimulated by the concentration gradient of this cytokine. CXCR7 is a second receptor for CXCL12 that removes CXCL12 from the extracellular space and destroys it in lysosomes. Elimination of CXCL12 by this receptor increases the CXCL12 concentration gradient, which maintains CXCR4 signaling and thus induces chemotaxis. Therefore, the expression of CXCR7 causes prolonged exposure of CXCR4 to CXCL12 and enhances metastasis and growth of breast cancer cells by sustaining the signaling of the mentioned pathway [11, 12].

Thiazolidinediones (TZDs) are pharmacological ligands of peroxisome proliferator-activated receptor gamma (PPARϓ) nuclear receptors [13]. Ligand-activated PPARϓ attenuates the function of many pro-inflammatory transcription factors including NF-κβ [14, 15]. CXCR7 and CXCR4 genes include promoters of the NF-κβ binding site; therefore, TZDs, including pioglitazone (PIO), are capable of inhibiting the invasion and metastasis of breast cancer cells as [16, 17]. PIO inhibits CXCR7 expression and macrophage chemotaxis by effecting PPARϓ [18]. On the other hand, pioglitazone reduces surface CXCR4 mRNA and protein in cancer cells. Therefore, targeting PPARϓ to suppress CXCR4 expression and ultimately inhibit metastasis is an approach to cancer treatment [19, 20]. Anthracycline like doxorubicin (DOXO) is commonly prescribed in the treatment of TNBC [21, 22]. Despite the antitumor effects of DOXO on primary tumors, DOXO can increase the risk of metastasis by increasing NF-κβ activity and CXCR4 expression [23]. Therefore, targeting CXCR4 would be a promising approach in reducing the invasion of cancer cells during chemotherapy. In this regard, doxorubicin and pioglitazone co-administration could be a promising approach to simultaneously downregulate CXCR4 expression and NF-κβ activity.

In this study, the effect of pioglitazone was evaluated on the doxorubicin-treated MDA-MB-231 breast cancer cell line. CXCR4 is upregulated in TNBC. This upregulation increases the activity of the CXCR4/CXCR7/CXCL12 pathway. Combination of PIO and DOXO seems to be beneficial as PIO can decrease the expression of CXCR4 and CXCR7 genes, which play a crucial role on metastasis and are upregulated in response to DOXO. To the best of our knowledge, this study was the first report on the effects of co-administration of PIO and DOXO on migration and invasion of breast cancer cells. The effect of PIO-DOXO co-administration on the expression of CXCR4 and CXCR7 genes and cell migration of the MDA-MB-231 breast cancer cell line was statistically analyzed in this study. The results of this study suggest a simple approach to reduce tumor cell migration and cancer metastasis by adding PIO to DOXO in the treatment of breast cancer.

## Material and methods

### Chemicals and reagents

Dulbecco’s modified Eagle medium/Nutrient Mixture F-12 (DMEM-F12), penicillin, streptomycin, and amphotericin were purchased from Gibco. Pioglitazone was purchased from Sigma (St. Louis, MO), and doxorubicin was purchased from Pharmacia Upjohn-Italy (Milan, Italy). Fetal bovine serum (FBS), phosphate-buffered saline (PBS), trypsin, ethylenediaminetetraacetic acid (EDTA), dimethyl sulfoxide (DMSO), and bovine serum albumin (BSA) were purchased from Merck (Germany). The MDA-MB-231 cell line was obtained from Iran Pasteur Institute (Tehran, Iran).

### Cell culture

The MDA-MB-231 breast cancer cell line was cultured in the DMEM-F12 medium containing 10% FBS, 100 U/ml penicillin, 100 μg/ml streptomycin, and 25 μg/ml amphotericin. Four control groups were prepared by treating cells with 200 and 1000 nM concentrations of PIO, 0.1% DMSO, while the control group was not exposed to any medication. Two main study groups consisted of (a) cell lines treated with either 200 nM or 100 nM DOXO and (b) cell lines treated with the combination of 1000 nM PIO and 100 nM DOXO. For a typical cell culture, 10^6^ cells were seeded in six-well plates containing a complete culture medium. Upon reaching 80% confluence, the cells were washed with PBS and were incubated in a culture medium for 24 h along with the mentioned concentrations of DOXO and PIO at 37 °C and 5% CO_2_. After 24 h, the cells were washed with PBS to remove DXO and were treated with the mentioned daily doses of PIO for 21 days. At this time, the cells were detached and repassaged using 0.25% trypsin-0.53 mM EDTA until reaching 80% confluence. On day 21, the cells of the main groups were again exposed to a 100-nM dose of DOXO. Subsequently, the doxorubicin-containing medium was washed, and the cells were cultured for 48 h in a culture medium before performing further tests. All samples were prepared in triplicates.

### RNA extraction and quantitative reverse transcription (RT-PCR)

To investigate the relative expression of CXCR4 and CXCR7, the RNA of cells from different study groups was extracted after the abovementioned treatment according to the extraction protocol provided by the Extraction Kit Manufacturer (Bio Basic, Canada). QRT-PCR was performed using SYBR Premix EX Taq II and the primers (Table 1). The reaction profile of qRT-PCR was applied to Rotor Gene 6000 system (Corbett Research, Australia) (Table 2). The relative expressions of the genes were determined using standard diagrams and the 2^−ΔΔct^ method with beta-actin as the reference gene [24]. The difference in relative expression of the genes was analyzed by fold change according to 2^−ΔCT^ treatment group/2^−ΔCT^ control based on the following formula:

$$\Delta \Delta \mathrm{CT}=\Delta \mathrm{CT}\ \mathrm{target}\ \mathrm{samples}\ \left[\mathrm{CT}\ \mathrm{target}\ \mathrm{gene}-\mathrm{CT}\ \beta\ \mathrm{actin}\left]-\Delta \mathrm{CT}\ \mathrm{control}\ \mathrm{samples}\ \right[\mathrm{CT}\ \mathrm{target}\ \mathrm{gene}-\mathrm{CT}\ \beta\ \mathrm{actin}\right]$$

**Table 1:** Information of the primers used in this study

Gene	Forward primer	Reverse primer	Product size (bp)
β-ACT	AGATCATTGCTCCTCCTGAG	AGTCATAGTCCGCCTAGAAG	159
CXCR4	ACAGTCAACCTCTACAGCAG	ATCCAGACGCCAACATAGAC	137
CXCR7	CCGGACGTCATTTGATTGC	TGAAGGAGAGCGTGTAGAG	180

**Table 2:** The RT-PCR reaction profile

Step	Time	Temperature (°C)	Cycles
Initial denaturation	30 seconds	95	1
Denaturation	5 seconds	95	40
Annealing	30 seconds	55	
Extension	45 seconds	72	
Final extension	5 minutes	72	1

### Surface marker analysis by flow cytometry

To examine the expressions of CXCR4 and CXCR7 surface receptors on MDA-MB-231 cells, flow cytometry was performed in all groups after treatment. To do this, 10^5^ cells from each group were trypsinized, washed twice with FACS buffer (PBS containing 2% BSA), and incubated for 45 min with phycoerythrin (PE)-conjugated antibodies against CXCR7 and CXCR4 (BioLegend, USA) at 4 °C. The cells were washed again with FACS buffer, and then, 4% paraformaldehyde was added to the cells for immobilization. PE isoantibodies (BioLegend, USA) were also used as controls. The expression of the cell surface receptors was measured by FACS Calibur flow cytometry (Becton Dickinson, USA). Flow cytometric data were analyzed using Flow Jo v10.5.3 software, and the differences in the expression of the markers were reported as mean fluorescent intensity.

### Wound scratch assay

Scratch assay evaluated the wound healing ability in the absence of stimulant material (FBS). For this purpose, 2 × 10^5^ cells from each group were cultured in 24-well plates for 24 h in a DMEM-F12 medium containing 10% FBS. After reaching 80% confluence, cells were cultured in the presence of mitomycin C (0.02 mg/ml) for 2 h to inhibit cell proliferation. The scratches were subsequently created with pipette tips. To evaluate cell migration, the wells were photographed by inverted light microscope within 0- and 24-h intervals and the percentage of filled area at 24 h relative to the hour zero was assessed in each group by ImageJ software.

### Migration assay

A Transwell kit with 8-μm pores was used to investigate the directed cell migration under the concentration gradient of stimulant (FBS for the main study groups). First, 25 × 10^3^ cells from each group were added to the upper chamber of the Transwell kit together with 50 μl of the FBS-free DMEM-F12 medium. Then, 300 μl of medium containing 10% FBS was added to the bottom chamber, and the cells were incubated for 18 h. After washing with PBS and staining the porous membrane with crystal violet, the average number of cells transferred in five fields was calculated using light microscopy.

### Statistical analysis

In this study, the Statistical Package for Social Sciences (SPSS) version 20 (IBM Corporation, Armonk, NY, USA) was used. The Mann-Whitney *U* test was used to compare gene and protein expression, as well as wound healing ability between DOXO-PIO-treated cells and the control group. The level of statistical significance was considered as *P* ≤ 0.05.

## Results

### Effect of pioglitazone-doxorubicin combination on CXCR4 and CXCR7 gene expression

**Fig. 1:**
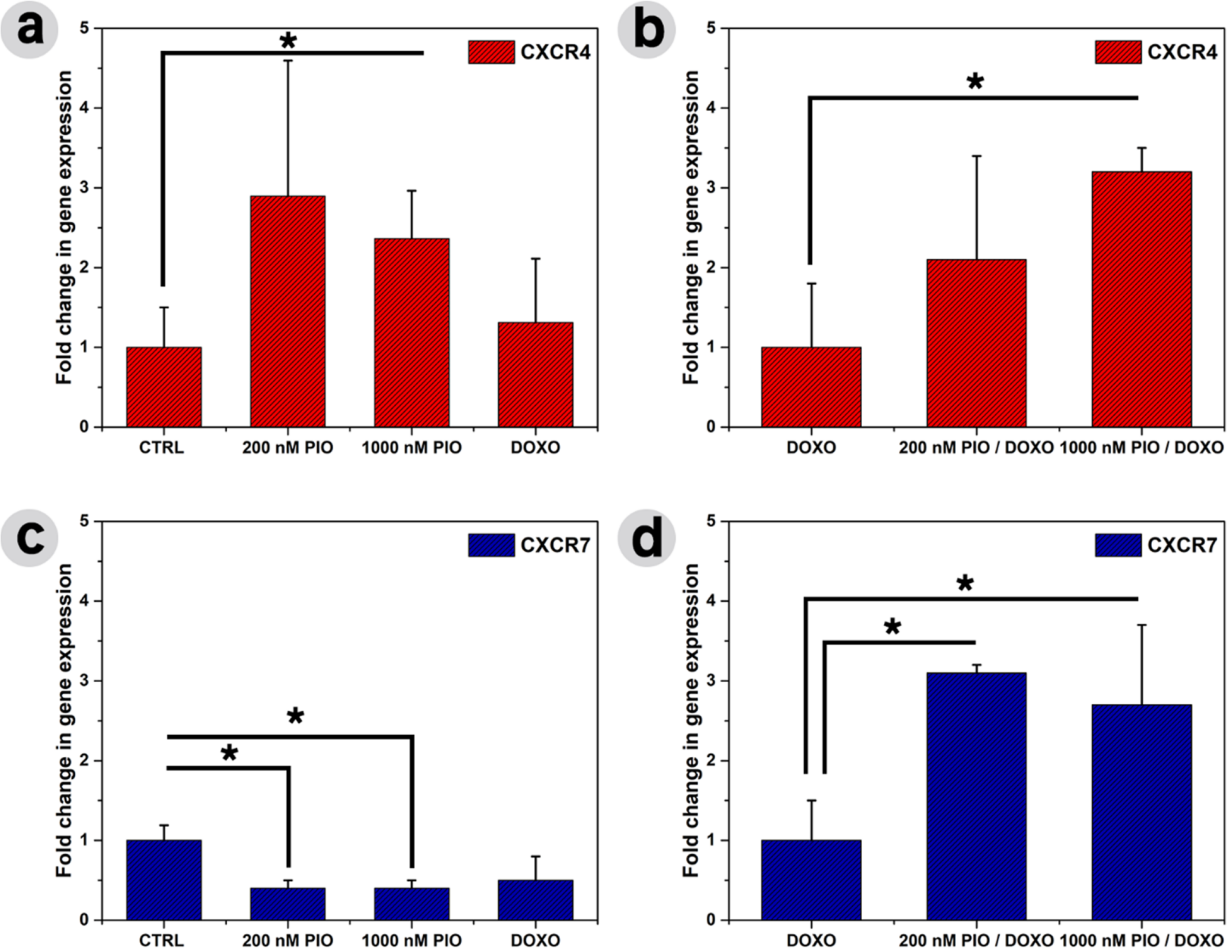
Evaluation of pioglitazone effects on CXCR4 and CXCR7 gene expression after 21 days. **a** The CXCR4 gene expressions among control groups as well as DOXO-treated cells (*P* = 0.049). **b** The CXCR4 gene expressions in cells receiving DOXO with each dose of PIO simultaneously which compared with cells treated with DOXO alone (*P* = 0.046). **c** The CXCR7 gene expressions among control groups as well as DOXO-received cells (*P* = 0.049). **d** The CXCR7 gene expressions in cells receiving DOXO with each dose of PIO simultaneously which compared with DOXO-treated cells alone (*P* = 0.049)

QRT-PCR and 2^−ΔΔct^ methods were used to assess independent and combined effects of PIO and DOXO on the expression of CXCR4 and CXCR7 genes in MDA-MB-231 cells. The results revealed that CXCR4 gene expression did not significantly change in the cells treated with a 200-nM concentration of PIO for 21 days compared to the control group, which did not receive any treatment (*P* > 0.05). However, the expression of the CXCR7 gene was decreased by 0.4 ± 0.07 fold (*P* = 0.049) in the PIO-DOXO combination group. On the other hand, treatment of cells with 1000 nM PIO was associated with a 2.3 ± 0.8-fold increase in CXCR4 gene expression and a 0.4 ± 0.08-fold decrease in CXCR7 expression (*P* = 0.049). The expression of CXCR4 and CXCR7 genes did not change significantly in the cell groups receiving 100 nM DOXO once at the beginning of treatment and again on day 21. Results of the main groups, which received two concentrations of PIO for 21 days with DOXO once at the beginning of treatment and again at day 21, indicated that the expression of the CXCR4 gene did not change in the group receiving 200 nM PIO plus 100 nM DOXO (*P* = 0.27), but CXCR7 gene expression significantly increased (by 3.15 ± 0.3 fold, *P* = 0.049) in the same group. Besides, CXCR4 and CXCR7 gene expression increased by 3.15 ± 0.3 and 2.77 ± 1.03 fold (*P* = 0.049), respectively, in the cells receiving 1000 nM PIO with 100 nM DOXO. Overall, simultaneous treatment of MDA-MB-231 cells with DOXO and PIO increased the expressions of CXCR4 and CXCR7 genes (Fig. 1).

### Effect of pioglitazone-doxorubicin combination on cell surface proteins

**Fig. 2:**
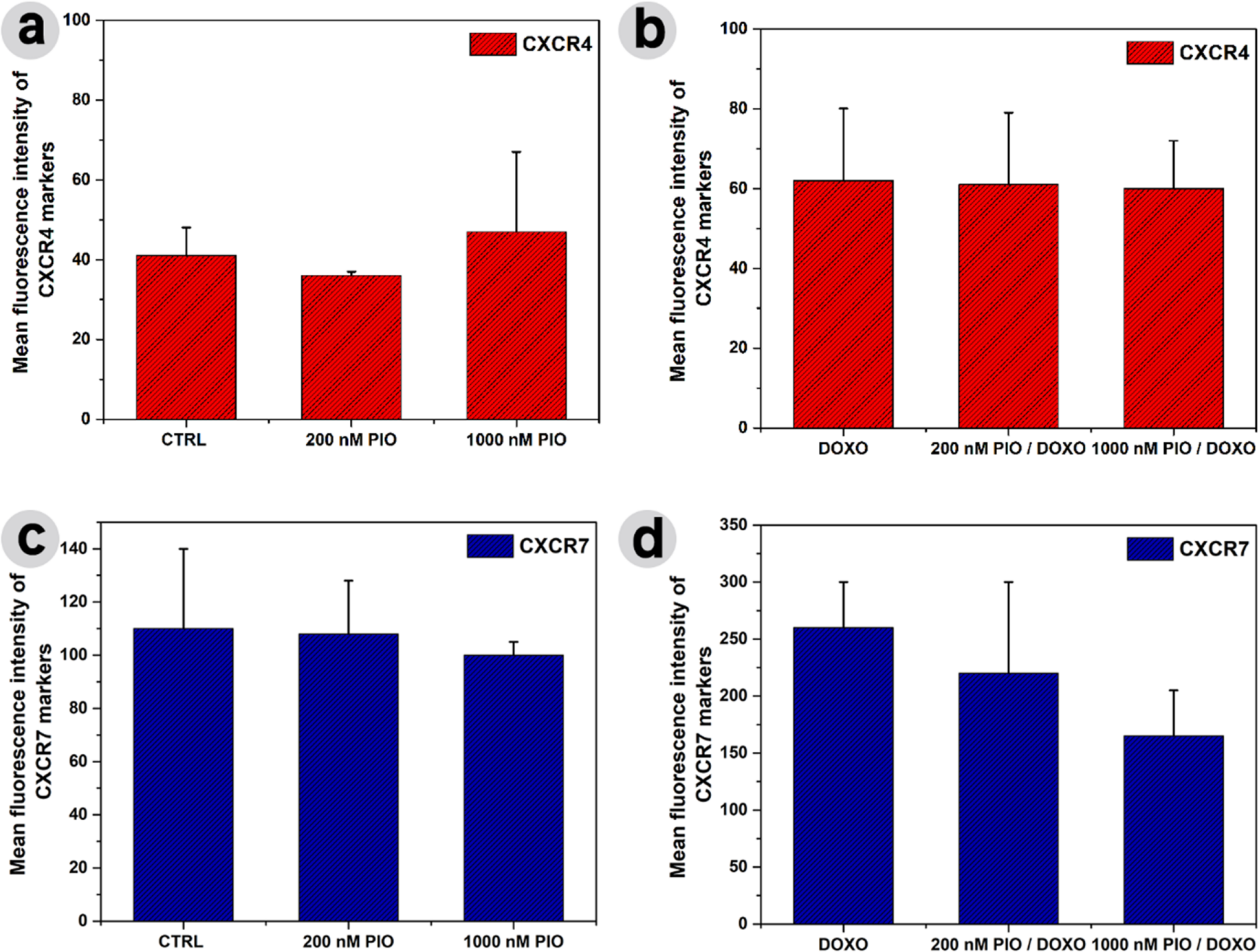
Results of CXCR4 and CXCR7 marker expressions on the cell surface by flow cytometry technique after 21 days of drug treatment. **a** The expression of CXCR4 markers on cells receiving 200 and 1000 nM PIO compared to control cells. **b** The CXCR4 markers expressed on cells receiving DOXO with each dose of PIO simultaneously and compared to DOXO-treated cells alone. **c** The expression of CXCR7 markers on control cells. **d** The CXCR7 markers expressed on main cells receiving DOXO and PIO simultaneously and compared to DOXO-treated cells alone (*P* > 0.05)

Flow cytometry was used to measure the expression of CXCR4 and CXCR7 cell surface receptors (Supplementary information). The expression of CXCR4 and CXCR7 proteins among the cell group that received 200 nM PIO alone was 36.33 ± 1.13 and 107.45 ± 21.99 MFI values, respectively. The expressions of CXCR4 and CXCR7 proteins in the group receiving 1000 nM PIO alone were 47.13 ± 19.98 and 98.8 ± 5.93 MFI values, respectively. However, no significant difference was observed in the expression of CXCR4 and CXCR7 proteins between the control group that did not receive any drug and the two groups that received 200 and 1000 nM concentrations (*P* > 0.05). The surface expression of CXCR4 and CXCR7 proteins in the cell group receiving 200 nM PIO plus 100 nM DOXO was 61.6 ± 16.63 and 216 ± 62.22 MFI values, respectively. The surface expression of CXCR4 and CXCR7 proteins in the group that received 1000 nM PIO plus 100 nM DOXO was 59.66 ± 11.66 and 168 ± 41.01 MFI values, respectively. However, the findings revealed that treatment of cells with both 200- and 1000-nM concentrations of PIO for 21 days together with the administration of 100 nM DOXO once at the beginning of the cell treatment and again on day 21 had no effect on the surface expression of these receptors compared to the group that received only 100 nM DOXO (*P* > 0.05) (Fig. 2).

### Effect of pioglitazone-doxorubicin combination on the cell migration ability

**Fig. 3:**
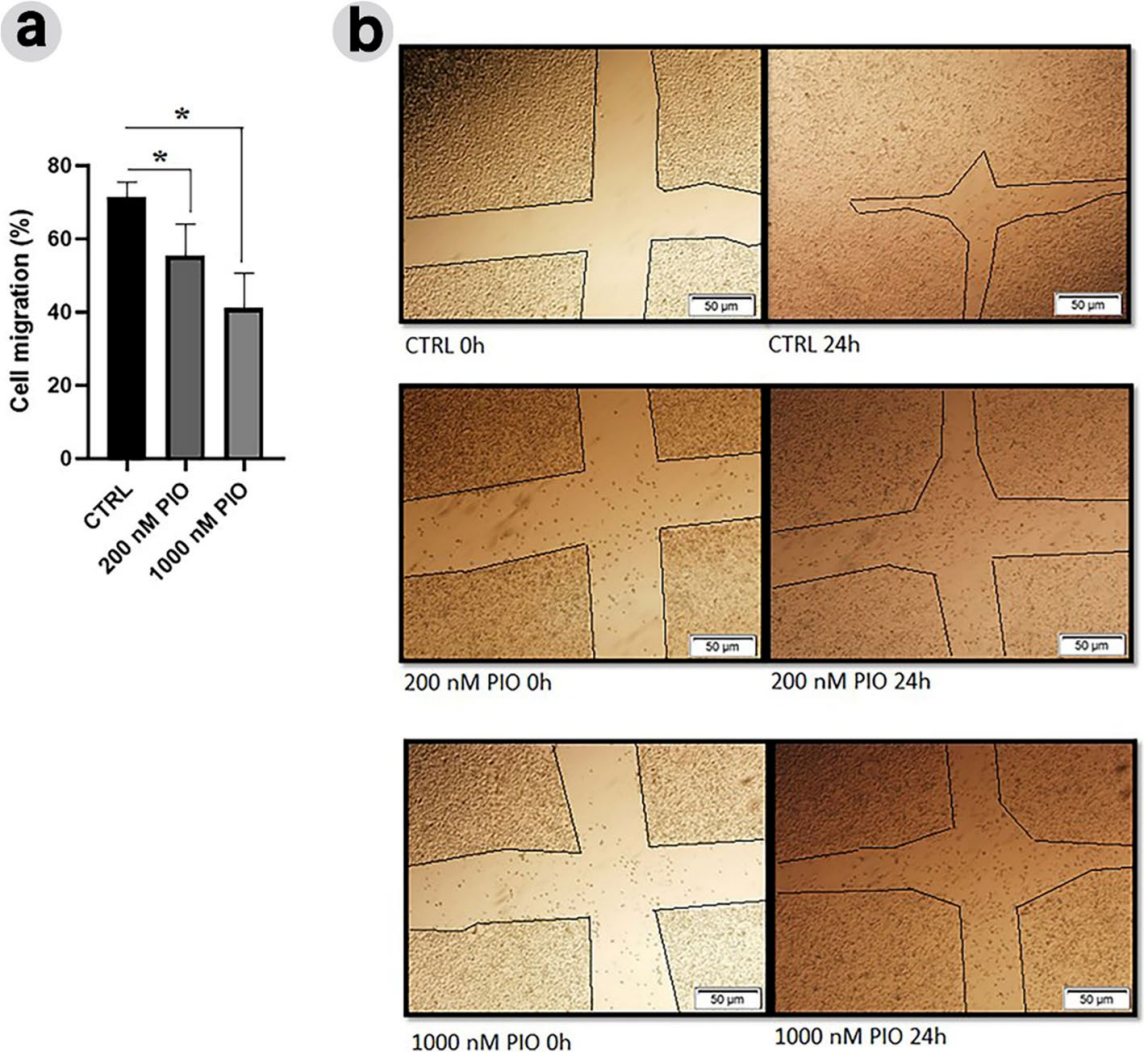
***a*** The percentage of the wound area filled by migrated cells after 24 h analyzed with ImageJ in groups treated with 200 and 1000 nM of PIO compared with the control group (*P* = 0.049). ***b*** The pictures of the wound areas after 24 h in cells treated with 200 and 1000 nM PIO compared to the control group

**Fig. 4:**
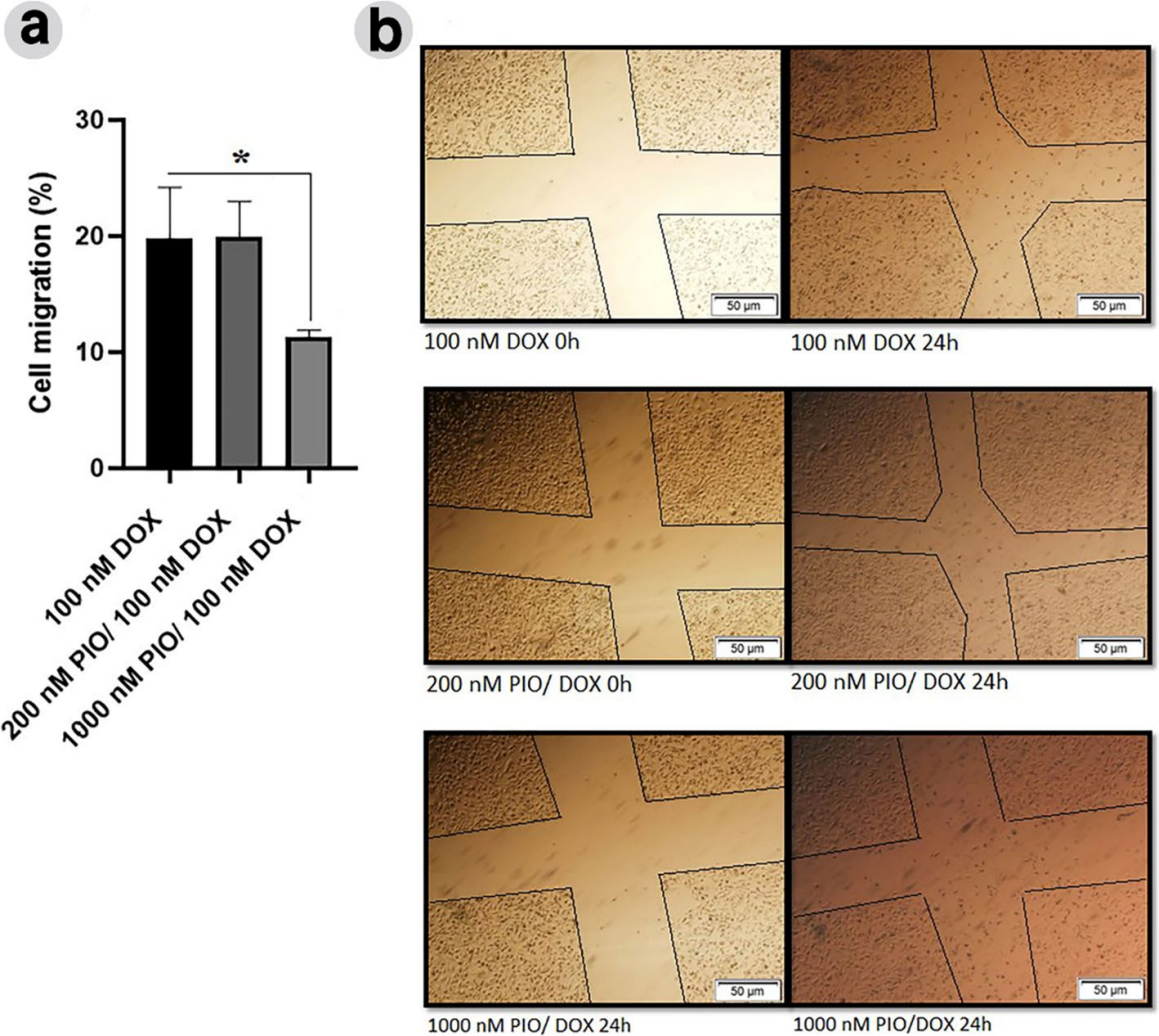
***a*** The percentage of the wound area filled by migrated cells after 24 h analyzed by ImageJ in groups treated with DOXO and each dose of PIO simultaneously (*P* = 0.049). ***b*** The pictures of the wound areas after 24 h in main groups of cells

The results of the wound healing technique showed that in the cells treated with 200 nM PIO alone, 55.4 ± 8.6% of the scratched area was filled after 24 h on the 21st day of treatment. This rate was 41.18 ± 9.4% in the group that was exposed to 1000-nM PIO concentration. Both concentrations of PIO alone reduced cell migration in the absence of a chemoattractant agent (FBS) compared to the control group that did not receive any drug (*P* = 0.049). Alternatively, simultaneous treatment of cells with 200 nM PIO and 100 nM DOXO led to 19.9 ± 2.9% filling of the scratched area, which was not significantly different from the group receiving 100 nM DOXO (*P* = 0.827). Nevertheless, 1000 nM PIO plus 100 nM DOXO filled only the scratched area by 11.3 ± 0.56% that indicates a significant decrease in cell migration compared to the DOXO control group (*P* = 0.049) (Figs. 3 and 4).

**Fig. 5:**
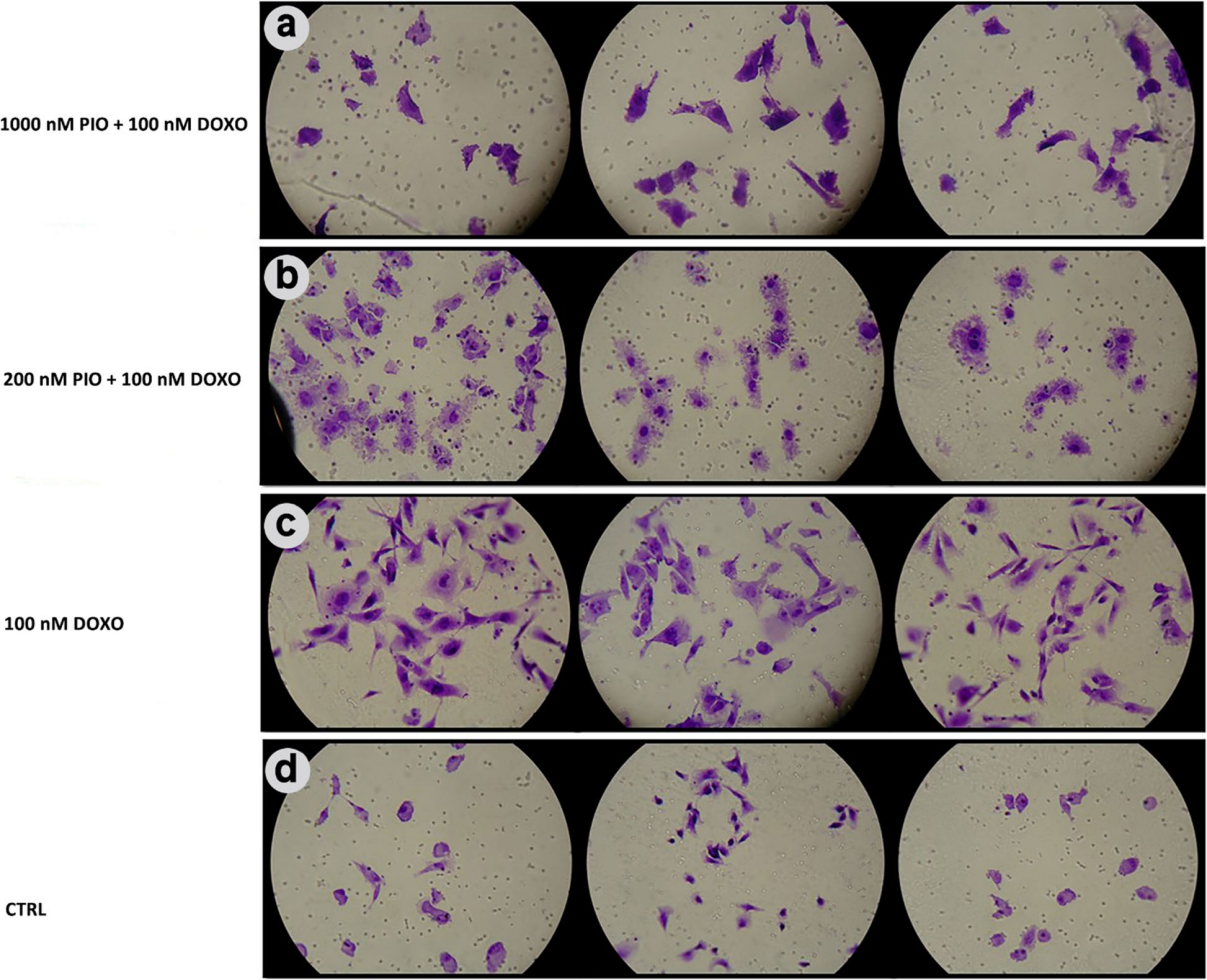
Results of Transwell migration assay under FBS concentration gradient after 18 h shows the number of migrated cells treated with **a** 1000 nM PIO and DOXO, **b** 200 nM PIO and DOXO, **c** 100 nM DOXO alone, and **d** the control group

The Transwell test and measurement of cell migration toward chemoattractant agent (10% FBS) were employed to evaluate and confirm these findings. The results showed that cell migration along the Transwell kit membrane significantly increased in MDA-MB-231 cancer cells that were exposed to 100 nM DOXO under FBS concentration gradient. On the other hand, the 1000 nM PIO plus 100 nM DOXO decreased the number of cells crossing the membrane as well as cell migration compared to the DOXO control group. The 200-nM concentration of PIO along with 100 nM DOXO caused a minor change compared to the DOXO control group (Fig. 5).

## Discussion

Triple-negative breast cancer is an advanced breast cancer with limited treatment options and a poorer prognosis relative to other types of breast cancer. The antitumor drug doxorubicin has been extensively used in the treatment of a wide range of cancers, including TNBC tumors [25, 26]. However, the use of doxorubicin is associated with increased cell migration and invasion. In the meantime, CXCR4/CXCL12 axis plays a crucial role in the migration and metastasis of cancer cells. Furthermore, CXCR7 as another receptor for CXCL12 is involved in the complex function of CXCR4. Previous researchers have suggested that the administration of anticancer medications like DOXO may enhance the invasion of tumor cells based on in vitro studies [27]. Studies have shown that DOXO treatment in ovarian cancer and doxorubicin-resistant non-small cell lung cancer (NSCLC) cells was associated with increasing CXCR4 gene expression [28, 29].

It is well-known that thiazolidinediones affect the function of several transcription factors, including NF-κβ, by binding to their PPARγ nuclear receptors. NF-κβ is a critical transcription factor for CXCR4 and CXCR7 genes; therefore, thiazolidinediones, including pioglitazone, decrease the expression of these genes by affecting NF-κβ activity [18, 30]. It is also believed that the activation of PPARγ by a thiazolidinedione called rosiglitazone suppressed the expression of the CXCR4 gene by recruiting a silencing mediator of retinoid and thyroid hormone receptor (SMRT), which is a co-suppressor to PPRE locus, in the CXCR4 gene promoter. This procedure reduces cancer cell migration and invasion [20]. Based on these findings, we aimed to investigate the effect of long-term exposure to PIO on the expression of CXCR4 and CXCR7 cell surface genes and proteins among doxorubicin-treated MDA-MB-231 cancer cells. QRT-PCR results showed that contrary to previous expectations and studies, PIO increased the expressions of CXCR4 and CXCR7 genes in doxorubicin-treated cells. In addition, a study by Choi et al. on colon cancer cells reported that low concentrations (< 1000 nM) of PIO significantly increased cancer cell proliferation and augmented *β*-catenin expression, thereby affecting the expressions of Wnt target genes [31], including CXCR4 [31, 32]. Therefore, in spite of the reports indicating a high expression of *β*-catenin in the MDA-MB-231 cell line, a 3-week chronic administration of low concentrations of PIO may affect the Wnt/*β*-catenin signaling pathway and increased CXCR4 gene expression independent of the PPARγ pathway [33]. This pathway was stated in another study that reported that thiazolidinediones depleted intracellular Ca2^+^ stores and inactivated eIF2α through phosphorylating independent of PPARγ [34].

According to the abovementioned findings, it can be inferred that chronic administration of PIO in our study inactivated eIF2α and affected the pathways involved in its translation and inhibition during treatment with DOXO. Furthermore, PIO had no effect on the expressions of CXCR4 and CXCR7 surface proteins despite the observed increase in the expressions of their genes. According to the results of the Transwell kit and scratch test, we showed that apart from increasing the expression of CXCR4 and CXCR7 genes, PIO decreased the migration in doxorubicin-receiving cells. These results were consistent with the reports from Hernandez et al. that the overexpression of CXCR4 along with CXCR7 reduced the invasion and destruction of the extracellular matrix [35]. As a scavenger receptor, CXCR7 drives CXCL12 into the cell and reduces its binding to CXCR4, thereby inhibiting CXCR4 signaling. This process is a mechanism for the suppression of chemotaxis towards low concentrations of CXCL12.

The molecules produced during cellular metabolism can promote tumor cell proliferation and metastasis. The uptake of nutrients and metabolic pathways in cancer cells differs from that of normal cells and provides cancer cells with the necessary metabolites to grow. UDP-glucose 6 dehydrogenase (UGDH), for example, converts UDP-glucose to UDP-glucuronic acid. UDP-glucuronic acid is essential for the production of polysaccharide molecules, including hyaluronic acid, that can activate cell surface receptors and initiate the EMT process. Alternatively, the inhibition of UGDH expression has been shown to decrease cell migration not due to reduction in UDP-glucuronic acid or hyaluronic acid levels but rather due to the accumulation of UDP-glucose in the cell. However, UDP-glucose accumulation reduces the stability of the mRNA that encodes the Snail transcription factor, which regulates the expression of EMT-related genes [36]. Snail plays a crucial role in the EMT process through direct binding to E-boxes located in the promoters of multiple genes involved in cancer cell metastasis, including Myosin-Va. MYO5A controls the cytoskeleton, cell morphology, and motility of filopodia [37]. Therefore, although PIO may have increased the expressions of CXCR4 and CXCR7 genes in concomitant treatment with DOXO, it delayed cell migration due to the accumulated glucose in the cells caused by pioglitazone-induced increased glucose and reduced expression of other proteins involved in the migration process.

In this study, PIO might have reduced cell migration in scratch test and Transwell kit by increasing the expression of the CXCR7 gene without affecting its expression if co-administered with DOXO. This hypothesis was consistent with a previous report that showed increasing T-cell chemotaxis despite low environmental concentrations of CXCL12 and decreased CXCR7 mRNA expression [38].

## Conclusions

In this study, the effect of PIO and DOXO co-administration was investigated on CXCR4 and CXCR7 gene expression in the MDA-MB-231 breast cancer cell line. It was observed that PIO was likely to decrease the motility of MDA-MB-231 cells via increasing the expressions of CXCR4 and CXCR7 genes and possibly through affecting other pathways associated with cell migration without affecting the expressions of surface proteins of these two receptors. In other words, chronic PIO treatment inactivated eIF2α and affected the pathways involved in its translation and inhibition during co-administration with DOXO. Accordingly, the synergistic effect of multiple anticancer therapy may reduce cancer cell migration, which may not be obtained by monotherapy.

## Supplementary Information

**Additional file 1: (**Geometric means of CXCR4 and CXCR7 cell surface expression in control groups and drug-treated groups).** Figure S1.** Flow Cytometry analysed geometric means of CXCR4 cell surface expression in three independent exposures of cells with drugs. **Figure S2.** Flow Cytometry analyzed geometric means of CXCR7 cell surface expression in two independent exposures of cells with drugs. **Figure S3.** Flow Cytometry analyzed geometric means of CXCR7 cell surface expression in two independent exposures of cells with drugs. **Figure S4.** Flow Cytometry analyzed geometric means of CXCR4 cell surface expression in three independent exposures of cells with drugs.

## Data Availability

The authors state that data and materials are available.

## Abbreviations

TNBC: Triple-negative breast cancer
TZDs: Thiazolidinediones
PPARϓ: Peroxisome proliferator-activated receptor gamma
PIO: Pioglitazone
DOXO: Doxorubicin
FBS: Fetal bovine serum
PBS: Phosphate-buffered saline
EDTA: Ethylenediaminetetraacetic acid
DMSO: Dimethyl sulfoxide
BSA: Bovine serum albumin
PE: Phycoerythrin
NSCLC: Non-small cell lung cancer
UGDH: UDP-glucose 6-dehydrogenase
