# Supplementary material for: Combined effects of pioglitazone and doxorubicin on migration and invasion of MDA-MB-231 breast cancer cells
Source: J Egypt Natl Canc Inst. 2022 Mar 28;34:13. doi: 10.1186/s43046-022-00110-x (PMC13314299; doi:10.1186/s43046-022-00110-x)
Supplement: Supplementary file 1 — Additional file 1: (Geometric means of CXCR4 and CXCR7 cell surface expression in control groups and drug-treated groups). Figure S1. Flow Cytometry analysed geometric means of CXCR4 cell surface expression in three independent exposures of cells with drugs. Figure S2. Flow Cytometry analyzed geometric means of CXCR7 cell surface expression in two independent exposures of cells with drugs. Figure S3. Flow Cytometry analyzed geometric means of CXCR7 cell surface expression in two independent exposures of cells with drugs. Figure S4. Flow Cytometry analyzed geometric means of CXCR4 cell surface expression in three independent exposures of cells with drugs. [file 43046_2022_110_MOESM1_ESM.docx]

**Supplementary Information**

**Combined effects of pioglitazone and doxorubicin on the migration and invasion of MDA-MB-231 breast cancer cells**


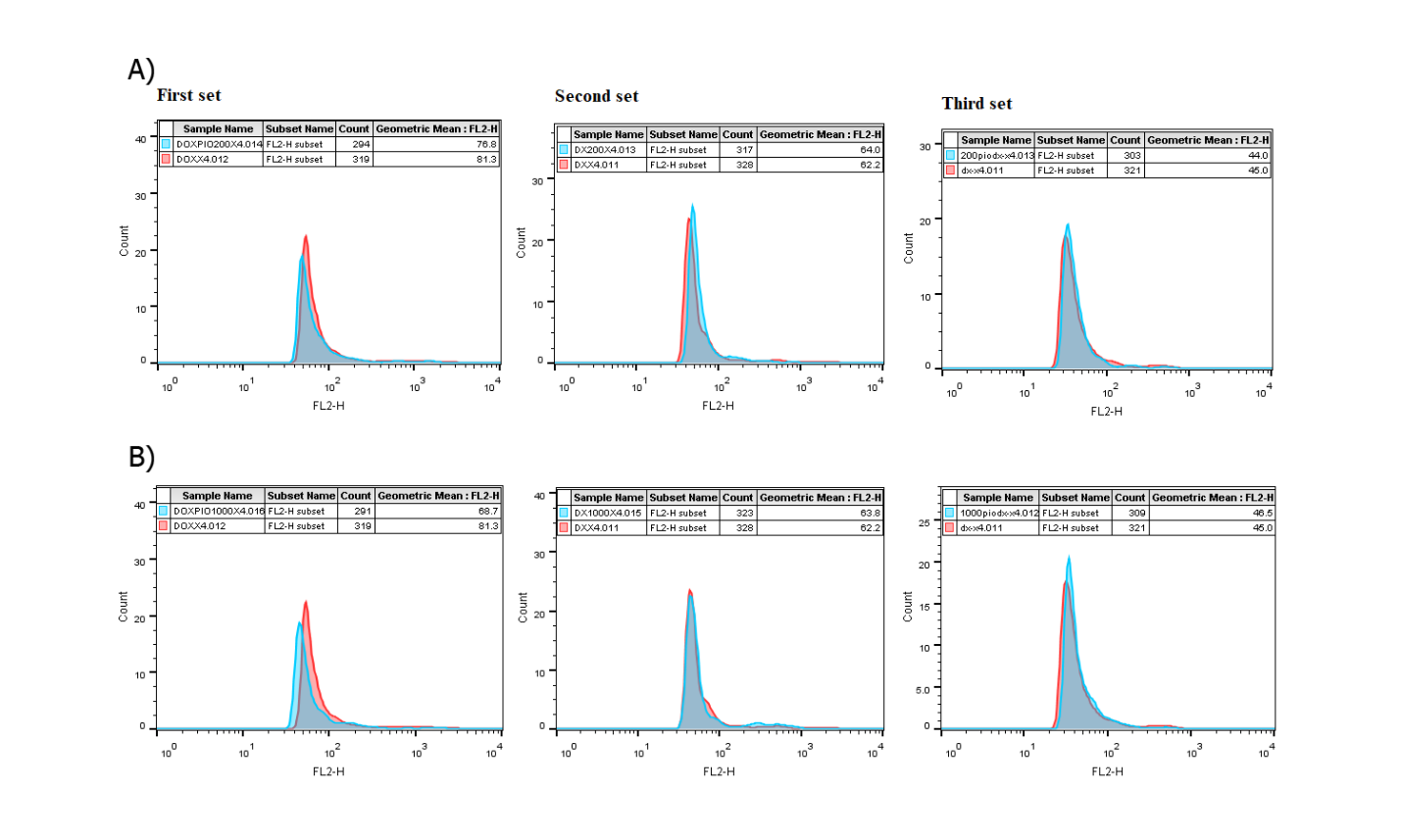


**Fig. S1.Flow Cytometry analysed geometric means of CXCR4 cell surface expression in three independent exposures of cells with drugs. The trends for geometric mean changes in cells receiving each PIO dose with DOXO simultaneously compared with DOXO-treated cells. Panel a) 200 nM PIO with DOXO treatment. Panel b) 1000 nM PIO with DOXO treatment.**

­­­­­­­
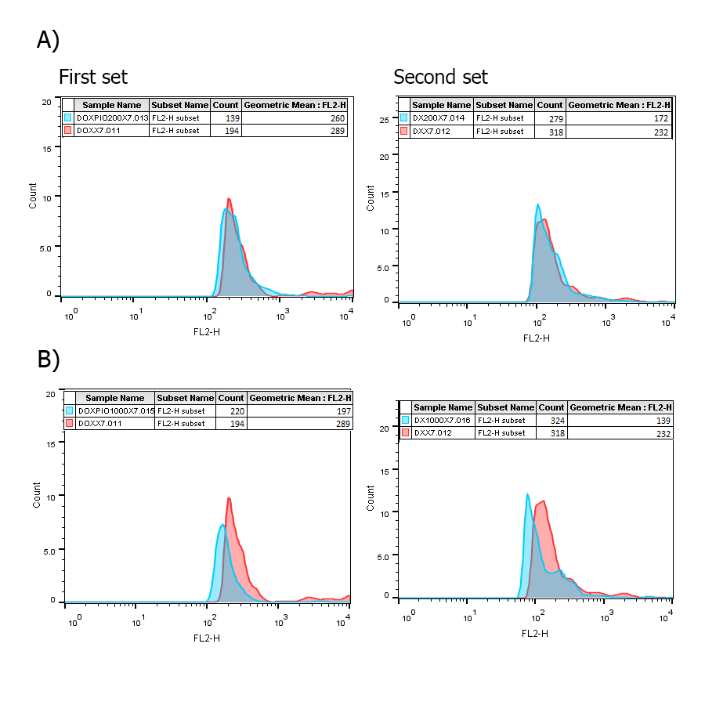


**Fig. S2. Flow Cytometry analyzed geometric means of CXCR7 cell surface expression in two independent exposures of cells with drugs. The trends for geometric mean changes in cells receiving each PIO dose with DOXO simultaneously compared with DOXO-treated cells. Panel a) 200 nM PIO with DOXO treatment. Panel b) 1000 nM PIO with DOXO treatment.**

­­­­­­­
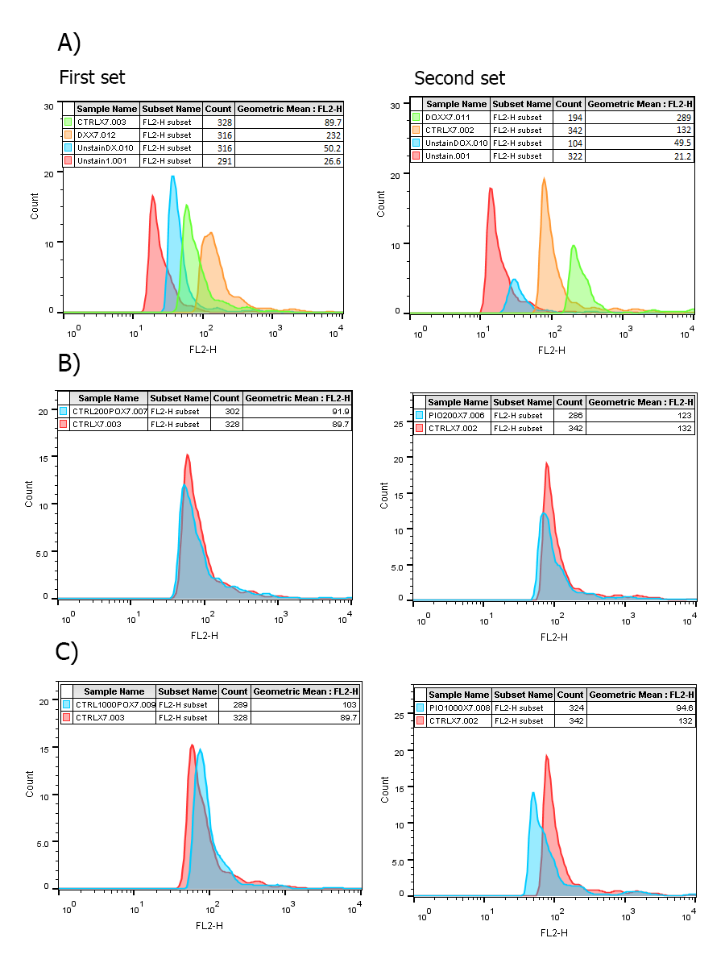


**Fig. S3. Flow Cytometry analyzed geometric means of CXCR7 cell surface expression in two independent exposures of cells with drugs. The trends for geometric mean changes in the group receiving each dose of PIO separately are compared with control cells and unstained cells. Panel a) Geometric means of cell treated with DOXO, control cells with PE-conjugated antibodies and DOXO-treated cell, control cells without PE-conjugated antibodies. Panel b) Comparison of 200 nM PIO-treated cell with the control group. Panel b) Comparison of 1000 nM PIO-treated cell with the control group.**

­­­­_
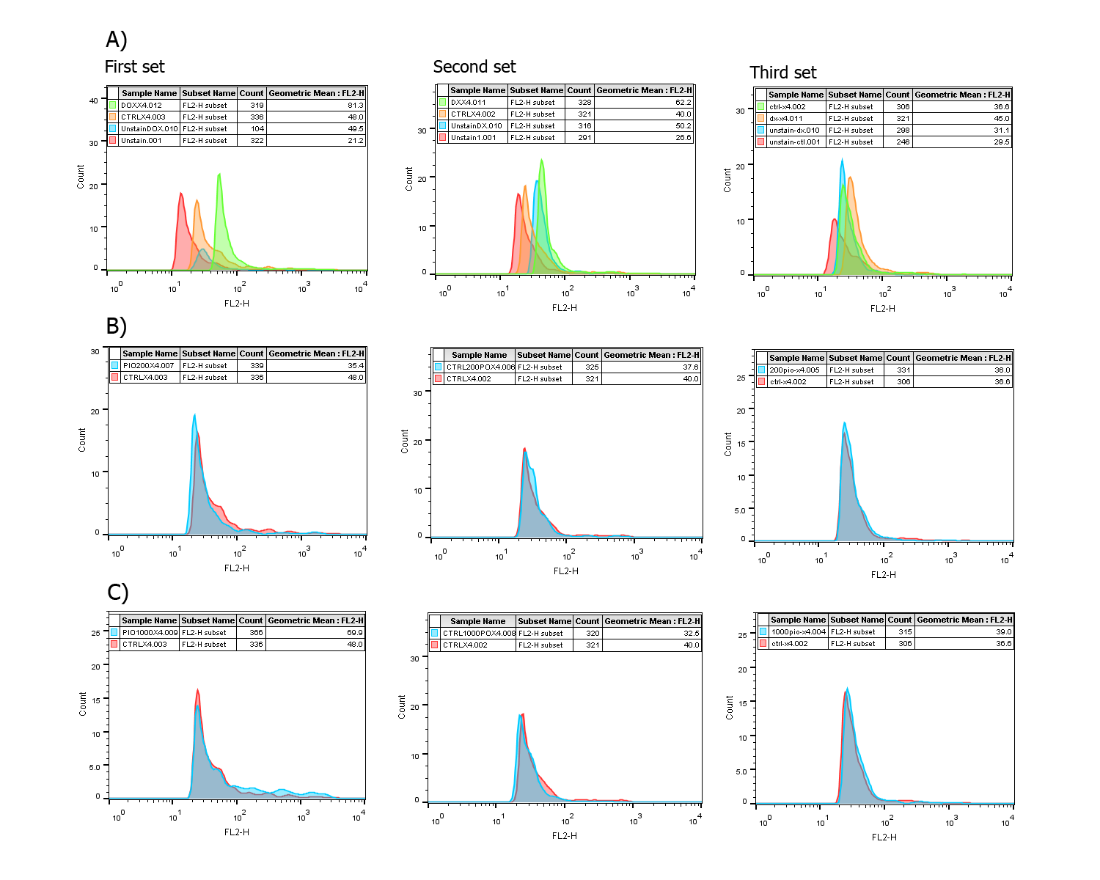
_

**Fig. S4. Flow Cytometry analyzed geometric means of CXCR4 cell surface expression in three independent exposures of cells with drugs. The trends for geometric mean changes in the group receiving each dose of PIO separately are compared with control cells and unstained cells. Panel a) Geometric means of cell treated with DOXO, control cells with PE-conjugated antibodies and DOXO-treated cell, control cells without PE-conjugated antibodies. Panel b) Comparison of 200 nM PIO-treated cell with the control group. Panel b) Comparison of 1000 nM PIO-treated cell with the control group.**
